# Effects of Drinking-Water Filtration on *Cryptosporidium* Seroepidemiology, Scotland

**DOI:** 10.3201/eid2001.120386

**Published:** 2014-01

**Authors:** Colin N. Ramsay, Adam P. Wagner, Chris Robertson, Huw V. Smith, Kevin G.J. Pollock

**Affiliations:** Health Protection Scotland, Glasgow, Scotland, UK (C.N. Ramsay, C. Robertson, K.G.J. Pollock);; University of Strathclyde, Glasgow (A.P. Wagner, C. Robertson);; International Prevention Research Institute, Lyon, France (C. Robertson);; Scottish Parasite Diagnostic Laboratory, Glasgow (H.V. Smith)

**Keywords:** fresh water, drinking water, Cryptosporidium, filtration, cryptosporidiosis, seroepidemiology, Scotland, bacteria

## Abstract

Continuous exposure to low levels of *Cryptosporidium* oocysts is associated with production of protective antibodies. We investigated prevalence of antibodies against the 27-kDa *Cryptosporidium* oocyst antigen among blood donors in 2 areas of Scotland supplied by drinking water from different sources with different filtration standards: Glasgow (not filtered) and Dundee (filtered). During 2006–2009, seroprevalence and risk factor data were collected; this period includes 2007, when enhanced filtration was introduced to the Glasgow supply. A serologic response to the 27-kDa antigen was found for ≈75% of donors in the 2 cohorts combined. Mixed regression modeling indicated a 32% step-change reduction in seroprevalence of antibodies against *Cryptosporidium* among persons in the Glasgow area, which was associated with introduction of enhanced filtration treatment. Removal of *Cryptosporidium* oocysts from water reduces the risk for waterborne exposure, sporadic infections, and outbreaks. Paradoxically, however, oocyst removal might lower immunity and increase the risk for infection from other sources.

## Abstract 2

Improved filtration is associated with reduced prevalence of antibodies against *Cryptosporidium* spp.

Each year since 2005, Health Protection Scotland has received reports of 500–700 laboratory-confirmed cases of cryptosporidiosis (10–14 cases/100,000 population/year); seasonality is usually markedly biphasic, peaking in spring and early autumn. Cryptosporidiosis is caused by >1 species/genotypes of the protozoan parasite in the genus *Cryptosporidium*, which infects a wide variety of animals including humans. The most common human pathogens are *Cryptosporidium hominis* and *C. parvum*. Characteristic signs of infection are profuse, watery diarrhea, often accompanied by bloating, abdominal pain, and nausea or vomiting. Illness is typically self-limiting but can last for 2–3 weeks; studies suggest an association with long-term health sequelae, such as reactive arthritis and postinfection irritable bowel syndrome (*1*,*2*). Moreover, severe, chronic diarrhea or even life-threatening wasting and malabsorption can develop in persons with severely compromised immune systems, particularly those with reduced T-lymphocyte counts, in the absence of immunotherapy (*3*).

Drinking water contaminated with *Cryptosporidium* oocysts is a recognized risk factor for human illness (*4*–*6*). Before or after treatment, water can be contaminated by a variety of sources, including livestock, feral animals, or humans (*7*). Oocysts can remain infectious in the environment for prolonged periods and are resistant to regular drinking-water disinfection treatments. For preventing human exposure, oocysts must be physically removed from water supplies; however, inadequate water filtration can expose persons to risk for infection from viable oocysts (*8*–*11*). 

Where drinking-water filtration has been enhanced to reduce oocysts counts, the incidence of reported clinical *Cryptosporidium* infection has been reduced (*6*,*11*). However, reported rates of infection are subject to variation, depending on factors such as local laboratory testing criteria, and exposure source attribution depends on the quality of risk-factor exposure data. Therefore, assessing trends in clinical infection rates might not be sufficiently sensitive for detecting changes in single-exposure risks. Variations in other risk factors (e.g., foreign travel, direct animal contact) can also obscure an effect associated with reduced exposure to oocysts in drinking water. Assessment of the effects of changes in environmental oocyst exposure would ideally be based on measuring population-level indicators, rather than relying on reported (self-selected) cases of laboratory-confirmed cryptosporidiosis. Alternatively, longitudinal variation in levels of antibody to *Cryptosporidium* oocyst proteins could be used to detect associations with variations in oocyst exposure.

The association between seropositivity and exposure to *Cryptosporidium* oocysts in drinking water has been investigated. Low levels of oocysts have been detected in 65%–97% of surface-water supplies, suggesting that many populations may be at risk (*12*). Elevated serologic responses have been detected in those whose drinking-water source is surface water rather than groundwater. The risk for oocyst exposure might therefore be higher for surface water than for groundwater sources (*13*–*15*), even after conventional filtration (*13*). However, chronic low-level exposure to oocysts in environmental sources, including drinking water, can stimulate protective immunity. Strong serologic responses to oocyst antigens have been associated with such environmental exposures (*16*,*17*).

To decrease the risk for waterborne *Cryptosporidium* infection from drinking-water supplies, the water industry established several barrier water treatment systems. In Scotland, water treatment has significantly reduced the concentration of *Cryptosporidium* oocysts in final (posttreatment) tap water. Before September 2007, however, the Loch Katrine system, which supplies the towns of Glasgow and Clyde, did not have such a filtration treatment. The risk from drinking unfiltered water was demonstrated in 2000, when an outbreak of cryptosporidiosis occurred among Glasgow residents living within the Loch Katrine supply area (*18*). To decrease this risk, in September 2007, enhanced treatment (rapid gravity filtration and coagulation) was introduced to the Loch Katrine supply system. This new system provided an opportunity to assess the public health effects of improving the standard of water filtration.

We investigated the prevalence of antibodies to the 27-kDa *Cryptosporidium* oocyst antigen among residents living in the Loch Katrine supply area (Glasgow) before and after the introduction of filtration and compared these with levels in a control population (Dundee) where no such change to drinking-water treatment occurred. Our main objective was to determine whether an association exists between prevalence of antibody response to the 27-kDa antigen and the standard of drinking-water treatment (filtered vs. unfiltered).

## Methods

### Study Sites and Populations

The study received approval from the Multi-Research Ethics Committee for Scotland and was conducted from April 2006 through October 2008. Volunteer blood donors were recruited in Glasgow (population 580,000; western Scotland) and Dundee (population 142,000; eastern Scotland). Each area receives drinking water from a separate surface-sourced system. Before September 2007, the Glasgow water supply received only rough screening treatment, but after that date, it was upgraded to match the specifications at Dundee (Clatto reservoir), the control system, which received rapid gravity filtration and coagulation. During the study period, no significant changes were made to the Dundee water treatment system, and no waterborne or other outbreaks of cryptosporidiosis were reported for either location. Blood samples for this study were collected during 4 periods (matched for seasonality): April–July 2006 (period 1), August–October 2006 (period 2), April–July 2008 (period 3), and August–October 2008 (period 4).

### Waterborne Oocyst Data

Potential exposure to waterborne oocysts was assessed by examining data on oocyst counts, which was routinely collected from the respective supplies for regulatory purposes. To reduce sampling bias, large sample volumes (≈1,000 L), collected before and after filtration, were used for oocyst detection. Filtamax filters (Genera Technologies, Newmarket, UK) were used for posttreatment water sampling; Cuno (3M, Bracknell, UK) filters were used for pretreatment water sampling because of their higher turbidity. 

### Blood Sample Collection and Donor Questionnaires

Blood donors from Glasgow and Dundee were informed of the aims of the study and asked to consent to participate for the duration of the project. At each blood donation session attended during the study period, volunteers were asked to confirm their agreement to allow a sample to be used for the study and to provide information about recent known exposure to potential risk factors for *Cryptosporidium* infection*. *Blood samples (1 mL) were sent in heparinized containers to the Scottish Parasite Diagnostic Laboratory for analysis.

### Western Blot 

Serum samples were analyzed by using immunoblot (mini-format) to measure IgG response to the 27-kDa antigen. The analytical methods are described elsewhere (*14*,*16*,*17*). However, because locally available human serum for use as positive control was insufficient, the positive control was derived from serum from a rabbit that had been immunized with a soluble lysate of *C. parvum* in Freund’s complete adjuvant.

Before each blood collection period, fresh rabbit control serum was prepared, aliquoted, and frozen. Serum samples (Dundee and Glasgow) from each collection period were tested in the same set of test runs, which enabled comparison of potential differences between the 2 geographic locations. Oocysts (Iowa isolate) were imported from the University of Arizona (Tucson, AZ, USA). The intensity of the serologic response to the antigen was digitally analyzed by using a Gel Doc 2000 Imaging System (Bio-Rad, Hercules, CA, USA). The intensity of each band was standardized by comparing the response intensity of each serum sample against a positive control (expressed as percentage positive response [PPR]). PPR standardization was performed by comparing the intensity of the study serum band with that of the positive control band from the same blot.

### Statistical Methods

To achieve a power of 95% for detecting a difference of at least 10% in seropositivity between prefiltration and postfiltration blood samples in the Glasgow cohort, 700 donors were required from Glasgow and 290 from Dundee. These numbers were based on results of the McNemar test and an assumption that ≈30% of the prefiltration samples would be seropositive. According to results of a standard normal test for differences in proportions, the power to detect a seropositivity difference between the cohorts of at least 10% would be >90%.

Several statistical methods, including univariate (χ^2^ or Fisher exact test) and multivariate techniques, simultaneously considered several explanatory variables and serologic values. The type of multivariate regression model used was dictated by the distribution of the response variables: linear regression for continuous (PPR) and logistic regression for binary (positive/negative) responses.

Each participant should have had serologic results from 4 periods, and results were expected to correlate with each other. To enable repeated observations from the same participants over time, we used mixed-effect regression models. These methods maximized the data at each period and accounted for new participants recruited to replace those lost to attrition.

### Measurement of Seropositivity

Antibody levels in immunized rabbits are probably higher than those in humans exposed to low levels of oocysts in the environment. Seropositivity was measured relative to the positive (rabbit serum) control (0 to >100% PPR). Some of the analyses used the actual PPR measurements; others required a binary measure (positive/negative response). For the latter analyses, a PPR level had to be selected to designate what constituted a positive response. Seropositivity can therefore vary, depending on the cutoff threshold value used to compare with the positive control.

Other studies of *Cryptosporidium* antibody seroprevalence that used serum from human patients rather than immunized rabbits have used a 20% cutoff threshold to designate positivity (*13*–*15*,*19*). In such studies, *Cryptosporidium* antibody seroprevalence has been 48%–76% of the study populations. Because our study used 20% PPR as the cutoff threshold, overall prevalence of antibodies against *Cryptosporidium* was similar for the entire study cohort (75%). Had the cutoff been increased to 30% PPR, then 64% of the entire cohort would have been designated as having a positive serologic response.

The choice of PPR cutoff threshold affected our ability to detect significant changes in the proportion of each cohort who were antibody positive. To determine what effect different PPR cutoffs would have on the analyses, we conducted a sensitivity analysis; as the PPR cutoff value increased, the chance of detecting a significant difference also increased.

### Multivariate Analysis of Risk Factors and Serologic Responses

Logistic models were fitted to the logistic response by dichotomizing the response at different PPR cutoff values; those >20% were designated as positive. Within the logistic models, we fitted a series of contrasts, which enabled us to test differences in serologic responses linked to differences in risk factor exposures between the cohorts and between collection periods. From the 8 main observation categories (2 cities and 4 periods), 7 comparisons (contrasts) were generated and used to assess variations in the proportions of persons with a positive serologic response. The main study hypothesis was that the introduction of filtration to the Glasgow supply would be associated with a change in seroprevalence levels in the Glasgow cohort. The prefiltration/postfiltration effect in Glasgow, compared with this effect in Dundee, was the major comparison used in the sensitivity analysis for choosing the optimal PPR cutoff for the main statistical analyses. Analyses of logarithmically transformed PPRs were used to identify relative differences across collection periods and city cohorts. We also compared the exposure risk profiles of those who had no (0) detectable responses with those who had some (>0) serologic responses by using χ^2^ tests.

Unless otherwise stated, we used R software version 2.80 (www.R-project.org) for the statistical analysis and Minitab statistical software version 14 (www.minitab.com) for information collation. In general, a significance level of 5% (p<0.05) was used for all analyses. However, because repeated tests were conducted for the same variable (the serologic response), the level used to assess statistical significance was corrected by using the Bonferroni correction. Because there were 31 demographic risk factor questions, a corrected significance level of 0.0016 (0.05/31), rather than the standard 0.05, was used.

## Results

### Study Participants

The original cohort consisted of 791 blood donors from Glasgow and 260 from Dundee. However, not all the original participants remained in the study, and some were replaced by new recruits (Table 1). The total number of study participants was therefore 1,437. Some participants did not donate blood during all 4 collection periods; others donated >1 time during some collection periods.

**Table 1:** Participants in study of antibodies against Cryptosporidium in drinking water, Scotland, UK, 2006–2008

Participants	Study period			
	1 (2006 Apr–Jul)	2 (2006 Aug–Oct)	3 (2008 Apr–Jul)	4 (2008 Aug–Oct)
Previous participants*	1,051	750	608	911
New recruits	0	133	253	0
Total	1,051	883	861	911
By city				
Glasgow	791	671	638	698
Dundee	260	212	223	213

*Donated blood at least 1 time since period 1.

### Oocyst Counts

Before September 2007, the oocyst detection rate in Clatto (final water) averaged 4.4 × 10^−4^ oocysts per 10 L (Table 2). During this period, consumers of Loch Katrine water were exposed to 13.2 × 10^−4^ oocysts per 10 L, which is 3 times higher. Therefore, the risk for waterborne infection was potentially greater for Glasgow residents. After filtration was introduced at Loch Katrine, the oocyst count decreased to zero in final water, representing complete removal of waterborne oocysts. During this period, consumers of Clatto water were also exposed to fewer oocytes (average 1.15 × 10^−4^ oocysts/10 L, reduced from the original 4.4 × 10^−4^ oocysts/10 L). Thus, both cohorts were exposed to reduced oocyst contamination in the drinking water, but the magnitude of reduction was greater for the Glasgow cohort (100% reduction) than for the Dundee cohort (75%). Speciated oocysts were *C. parvum, C. bovis, C. ubiquitum,* and other environmental genotypes. However, *C. hominis* was never isolated from either drinking water supply during the study period.

**Table 2:** Concentration of oocysts in drinking water before and after September 2007 installation of filtration system in Glasgow, Scotland, UK

Location, period	Concentration of oocysts/10 L		Reduction, %
	Raw	Final	
Dundee			
Before	0.000850	0.000439	48
After	0.001979	0.000115	94
Glasgow			
Before	0.004219	0.001320	69
After	0.000855	0.000000	100

### Questionnaire Responses

Few statistically significant differences in demographic or exposure risk factor variables were found between the 2 cohorts (Table 3). The relative similarity in demographic and risk factor profiles between the Glasgow and Dundee cohorts indicated that the populations were comparable.

**Table 3:** Demographic characteristics of participants in study of antibodies against Cryptosporidium in drinking water, Scotland, UK, 2006–2008*

Characteristic	Location, water filtration status			
	Dundee		Glasgow	
	Before	After	Before	After
Mean age, y	42.3 (40.8–43.9)	45.5 (43.8–47.2)	38.8 (37.9–39.6)	42.7 (41.7–43.6)
Female, %	44.2 (38.3–50.3)	45.2 (38.7–52)	47.1 (43.9–50.3)	43.1 (39.6–46.8)
Swam in past 12 mo, %	64.6 (58.6–70.2)†	48.3 (41.6–55.1)‡	58.4 (55.1–61.5)†	50.1 (46.4–53.7)‡
Have with <5 children, %	8.5 (5.7–12.5)†	9.5 (6.2–14.3)‡	7.4 (5.9–9.2)†	7.5 (5.8–9.7)‡
Have pets, %	43.8 (37.9–49.9)†	38.8 (32.4–45.5)‡	32.3 (29.3–35.4)†	30.8 (27.5–34.3)‡
Drink unboiled water, %	96.2 (93.1–97.9)†	95.2 (91.4–97.4)‡	89.2 (87.1–91.1)†	88.4 (85.8–90.5)‡
Drink bottled water, %	69.2 (63.4–74.5)1	63.2 (56.4–69.4) ‡	77.4 (74.6–80)†	73.7 (70.3–76.8)‡

*Before, before filtration in Glasgow (periods 1 and 2); after, after filtration in Glasgow (periods 3 and 4). †Percentage of persons who answered “yes” to the question at period 1, period 2, or both. ‡Percentage of persons who answered “yes” to the question at period 3, period 4, or both.

During the first period, more Glasgow participants consumed bottled water and fewer consumed unboiled drinking water than did their Dundee counterparts (p<0.001). Fewer participants in each cohort reported swimming after September 2007 than before (p = 0.011 and p = 0.015, respectively).

### Serologic Responses

According to univariate analyses, the only significant difference was that participants who had no (0) antibody response were significantly younger (p<0.001) than those with a detectable (>0) serologic response. The importance of age to participant serologic response was also assessed by using a linear model fitted to the square root of the serologic response (to normalize the distribution). In this model, age was associated with a statistically significant difference in the serologic response (p<0.0001); for each additional year of age, the serologic response increased by ≈0.35%.

In this study, serologic response to *Cryptosporidium *oocysts was positive for 75% of participants in both cohorts. The mean serologic response showed an overall increase over the 3-year period, as measured by the proportion with a serologic response (positive/negative, logistic model) and the average PPR (linear model) in both cohorts (Figure). 

**Figure:**
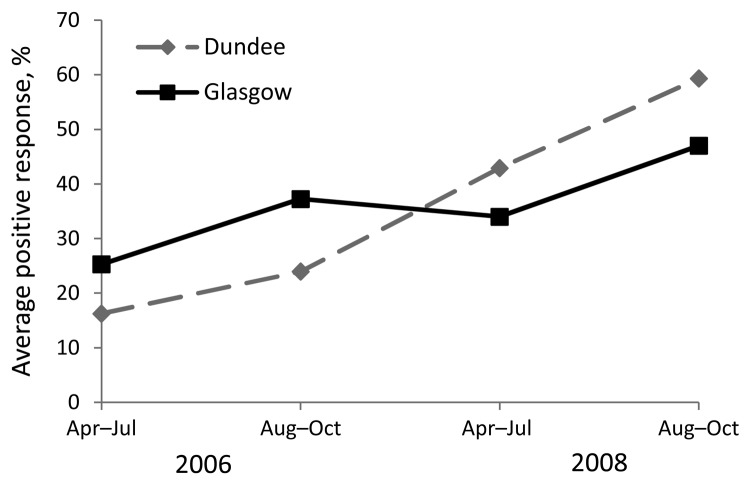
Mean percentage positive response (PPR) (IgG) to the 27-kDa antigen of *Cryptosporidium* oocysts among blood donors in Glasgow and Dundee, Scotland, 2006–2008. This graph represents the (model estimate) geometric mean PPR for an average participant followed up for the 4 time periods. The plot does not represent the proportion of participants for whom serologic response to the 27-kDa antigen was positive.

Both cohorts showed an overall increase over the whole 3-year period, as measured by the proportion with a serologic response (positive/negative, logistic model) and by the average PPR (linear model) (Figure). For periods 1 and 2, the responses for the Glasgow cohort were higher than those for Dundee. However, for the Glasgow cohort, the average serologic response (PPR) for period 3 dropped below that for period 2. This finding coincided with the introduction of enhanced filtration treatment at Loch Katrine in September 2007. The Glasgow response during period 3 was also lower than the Dundee response during period 3. Although for period 4, the average serologic response among participants in both cohorts again increased, the mean response for Glasgow remained lower than that for Dundee. Further analysis of the data, with the second linear mixed effects model fitted to the log of the PPR, indicated that mean serologic responses for Glasgow participants decreased by 32%, compared with those for Dundee participants, after enhanced filtration began at Loch Katrine (Table 4). This step-change reduction was statistically associated with introduction of the new treatment.

**Table 4:** The coefficients and significance of the terms used in the second linear mixed effects model fitted to the log of the percentage positive response*

Model element	Coefficient		Coefficient exponentiated (95% CI)	% Difference (95% CI)
	Value (95% CI)	p alue		
Intercept	2.79 (2.61 to 2.98)	0.0000	16.32 (13.57 to 19.63)	NA
City†	0.09 (0.02 to 0.15)	0.0077	1.09 (1.02 to 1.17)	19 (5 to 33)
Pre/post filtration periods‡	0.29 (0.24 to 0.33)	0.0000	1.33 (1.27 to 1.4)	66 (54 to 79)
Period 2 vs. period 1§	0.16 (0.1 to 0.22)	0.0000	1.17 (1.1 to 1.25)	34 (20 to 49)
Period 4 vs. period 3¶	0.12 (0.06 to 0.19)	0.0004	1.13 (1.06 to 1.21)	26 (11 to 42)
Pre/postfiltration, Glasgow vs. Dundee#	−0.17 (−0.22 to −0.12)	0.0000	0.84 (0.8 to 0.88)	−32 (−40 to −23)
Period 2 vs. period 1, Glasgow vs. Dundee**	0.06 (0 to 0.12)	0.0690	1.06 (1 to 1.13)	12 (−1 to −26)
Period 4 vs. period 3, Glasgow vs. Dundee††	0.07 (0 to 0.14)	0.0475	1.07 (1 to 1.15)	14 (0 to 29)
Minimum donor age‡‡	0.02 (0.01 to 0.02)	0.0000	1.02 (1.01 to 1.02)	NA

*Calculated for study of antibodies against Cryptosporidium spp. in drinking water, Scotland, 2006–2009. NA, not applicable.  †Negative value implies fewer/lower serologic responses in Glasgow than in Dundee, averaged over the 4 periods.  ‡Positive value implies more/higher responses in periods 3 and 4 compared with periods 1 and 2, averaged over the periods and cities. Periods 3 and 4 are post filtration in Glasgow.  §Positive value implies more/higher responses during period 2 compared with period 1, averaged over the cities.  ¶Positive value implies more/higher responses during period 4 compared with period 3 averaged over the cities.  #Negative value implies fewer/lower responses after filtration (period 3 and 4) at Glasgow than at Dundee. This is a comparison of the change in response from periods 1 and 2 with periods 3 and 4 in Glasgow with the same change in Dundee. **Positive value implies more/higher change in responses at Glasgow than at Dundee in period 2 compared with period 1.  ††Positive value implies more/higher change in responses at Glasgow than at Dundee in period 4 compared with period 3.  ‡‡Earliest age at which a sample was collected from a donor.

The mixed regression analysis of other risk factors for exposure to *Cryptosporidium* oocysts identified 4 positive findings, although none was statistically significant (by Bonferroni correction). Average serologic response to *Cryptosporidium* oocyst antigen was lower among participants who owned pets than among those who did not (p = 0.034), higher among those who had been swimming in the United Kingdom (p = 0.091), higher among those who consumed water from private supplies (p = 0.087), and lower among those who drank bottled water (p = 0.051).

Using the interactions model, we investigated the effect of introducing filtration in Glasgow and how this was associated with the sources of water consumed. The coefficients of all of these potential interactions were negative, providing evidence that enhanced filtration at Loch Katrine reduced serologic responses among all participants in Glasgow, regardless whether they drank only unboiled tap water, only bottled water, or both. Serologic response reduction was largest among participants who consumed any bottled water (either solely or in combination with unboiled tap water). However linear, quadratic, and cubic analyses indicated no evidence of a direct statistically significant association between the reported quantity of bottled water consumed and the reduced serologic response (i.e., no dose–response effect).

## Discussion

Opportunities to study the public health effects of major infrastructure changes are uncommon. Blood donors provide a convenient sample; they are relatively healthy, accessible, and generally cooperative. However, because they are predominantly younger to middle-aged adults, they are not completely representative of the population. Hence, the results of this study might be less applicable to children or elderly persons.

In this survey, serologic response to the 27-kDa *Cryptosporidium* oocyst protein was detected in 64%–75% of the total cohort. These findings are consistent with those from other populations served by surface-water sources (*14*,*19*). However, the aspects of this study that advance previous knowledge are as follows: the study involved a large number of participants in distinct cohorts followed over a considerable period, there was a defined intervention in one population but not the control population, and we sought to collect data on demographics and changes to biologically plausible risk factor exposures each time a participant donated a blood sample. The prospective cohort study design is a relatively robust epidemiologic method; high participant numbers enabled robust statistical analyses.

We were primarily interested in evidence of serologic response to oocyst exposure spanning a long period, as opposed to recent, acute exposure. Other serologic studies have assessed IgG serologic responses to the 15/17-kDa complex and the 27-kDa protein (*13*–*15*,*17*,*19*). After exposure to *Cryptosporidium *oocysts, a serologic response to both of these antigen groups usually peaks 4–6 weeks later (*20*). The 15/17-kDa marker declines to baseline levels in 4–6 months, but the 27-kDa marker remains elevated for at least 6–12 months. Because the 27-kDa response is considered a reliable marker for exposure to *Cryptosporidium *oocysts, we do not believe that our not investigating serologic responses to the 15/17-kDa complex devalues our findings.

 The Western blot method compared each serum sample with a positive control. Ideally, the positive control serum would have been derived from clinically ill persons, but because we did not have access to enough such serum to compare with >3,700 participant samples, we used rabbit-derived positive control serum. Although this form of calibration is recognized as acceptable for serologic studies, it is a possible limitation to this study despite the fact that our primary focus was detecting evidence of low level oocyst exposure, not confirming a clinical diagnosis of infection.

By using several statistical models, we detected a marked and dramatic step-change reduction in the seroprevalence of antibodies against *Cryptosporidium* among blood donors from the Loch Katrine water supply area after introduction of enhanced water filtration. We detected no corresponding step-change in seroprevalence among the control (Dundee) participants. The collated evidence suggests that this effect was mainly, if not solely, attributable to the introduction of filtration to the Loch Katrine water supply.

Given the fact that the serologic response levels (PPR) continued to increase after the oocysts were eliminated from the Loch Katrine water source, this study supports evidence that oocysts from other environmental sources stimulate background immunity levels. Exposure to oocysts through other sources (e.g., animal contact and contaminated food) might be at least as common as exposure through contaminated drinking water and might be more likely to transmit a higher dose of oocysts.

The study demonstrated that serologic responses to *Cryptosporidium* oocyst exposure were more likely (although not statistically significantly) to be higher among participants who ingested water while swimming in an indoor swimming pool or drank tap water from a private supply than among those who did not. The protective effect of antibody levels induced by such exposures is unknown; clinical cases of cryptosporidiosis have also been associated with swimming pools and private water supplies (*21*,*22*). More frequent use of water for recreational and other purposes might therefore increase the overall level of oocyst exposure, but it might also confer some resistance to infection on a per-event basis (*23*).

The observation in this study that age correlates with increased serologic response to *Cryptosporidium* antigen has been observed among persons with gastrointestinal and other infections (*24*–*26*). Children are more susceptible to gastrointestinal infection (including cryptosporidiosis) than adults, partly because adults have higher serum/mucosal antibody levels induced by the number of pathogen exposures during their lifetime (*15*,*26*).

Because partial (probably protective) immunity develops among persons with previous or ongoing exposure to *Cryptosporidium* oocysts, contamination of drinking-water sources might not necessarily manifest itself as detectable cases or outbreaks among local residents, but it might affect casual consumers more. Increased rates of clinical disease might not be the inevitable result of ongoing chronic low-level contamination of the water supply (*4*). The apparently complete removal of *Cryptosporidium* oocysts from drinking water supplied by Loch Katrine might have decreased the risk for waterborne illness. However, this reduction of low-level immune system stimulation might have paradoxically increased risk for infection from other sources of exposure.
